# Magnetic Tunnel Junction Applications

**DOI:** 10.3390/s20010121

**Published:** 2019-12-24

**Authors:** Nilson Maciel, Elaine Marques, Lírida Naviner, Yongliang Zhou, Hao Cai

**Affiliations:** 1 LTCI, Télécom Paris, Institut Polytechnique de Paris, 91128 Palaiseau, France; ecrespo@telecom-paris.fr (E.M.); lirida.naviner@telecom-paris.fr (L.N.); 2 National ASIC System Engineering Center, Southeast University, Nanjing 210096, China; zhouyongliang@seu.edu.cn (Y.Z.); hao.cai@seu.edu.cn (H.C.)

**Keywords:** magnetic tunnel junction, spin transfer torque, spin–orbit torque, voltage-controlled magnetic anisotropy, magnetic random access memory

## Abstract

Spin-based devices can reduce energy leakage and thus increase energy efficiency. They have been seen as an approach to overcoming the constraints of CMOS downscaling, specifically, the Magnetic Tunnel Junction (MTJ) which has been the focus of much research in recent years. Its nonvolatility, scalability and low power consumption are highly attractive when applied in several components. This paper aims at providing a survey of a selection of MTJ applications such as memory and analog to digital converter, among others.

## 1. Introduction

The discovery of Giant Magnetoresistance (GMR) [1,2] led to the development of spintronics [3]. Studies in this area have resulted in several important advances. With CMOS downscaling, the need of viable alternatives for reducing the leakage of power increases and spin-based devices appear as one of the most promising approaches to deal with this issue.

Magnetic Tunnel Junction (MTJ) has a significant role in the spintronics development [4]. Its structure has ferromagnetic layers connected with a insulator layer between them. MTJ has excellent scalability, low power consumption and potentially infinite endurance. In addition, unused MTJs can be completely powered off without the loss of data resulting in saving energy which render the MTJ suitable for several applications [4] such as memory devices and analog to digital converter. MTJ-based non-volatile memories (NVMs) have shown superior performance with respect to many relevant figure of merits such as energy efficiency and endurance [5]. MTJ-based memories allow for a ten year retention time operating at extremely low energy levels making them appropriate for batteries or low powered applications as internet of things (IoT) applications, specifically for sensor nodes located in difficult environmental conditions [6].

MTJ applications can also be found in several domains where data require processing and storage [7,8,9,10]. In radio frequency, spectrum-optimizing applications based on compressive sensing [11] desire to reduce the area of their circuits and decrease power consumption. In order to do this, MTJs can be used in mixed signal applications, such as comparators and analog to digital converters [9,10].

Recently, relevant progress has been done in MTJ research and it is expected to develop even further into commercial products in the near future. In order to help engineers and researchers who are just starting to work with MTJs or who are interested in learning about its wide and diverse application fields, this paper reviews important MTJ works reported in the literature over the last years.

The rest of the paper is organized as follows. Section 2 briefly explains the MTJ while also presenting STT, VCMA and SOT. The MTJ’s properties allows it be applied in several structures. Some of the most important digital applications are discussed in Section 3. On the other hand, Section 4 addresses some mixed and analog applications where MTJs can be found. Finally, Section 5 concludes the work.

## 2. Magnetic Tunnel Junction (MTJ)

**Figure 1:**
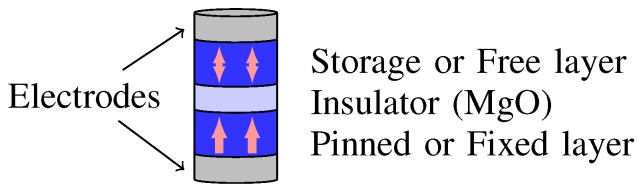
Magnetic tunnel junction (MTJ).

Figure 1 illustrates the basic MTJ structure. It consists of two ferromagnetic layers separated by the insulator layer MgO. The reference layer has unchangeable magnetization direction, while the magnetization direction can be changed in the free layer. Therefore, the magnetic field determines electrical properties of the MTJ. For applications, the difference of the conductance, resulted from the variations of the magnetic field in the ferromagnetic layers, is employed. The magnetization orientations ($m_{z}$) of the two ferromagnetic layers are related to a level of the MTJ resistance: low-resistance $R_{P}$ at a parallel state and high-resistance $R_{AP}$ at an anti-parallel state. With these two stable states of the MTJ, it can be easily used to represent logic 0 or logic 1 [12,13,14,15].

In order to control the electrical characteristics of MTJs, some methods have been developed for switching their stable states. The three main MTJ magnetization switching mechanisms are discussed below.

### 2.1. Spin Transfer Torque (STT)

Spin transfer torque was presented in Reference [16] as an alternative to improve the density of the first proposed MTJ circuits. The STT effect allows switch the MTJ state by a bidirectional current *I* when the current is bigger than a critical current $I_{c0}$. It improves the scalability of the circuit with MTJs allowing a denser layout and a simpler design due the use of the same line to write and read the MTJ state. However, using the same path can lead to unexpected writing when reading is in progress. Another disadvantage of the STT is that the current required to switch the states of the MTJ is not symmetrical (going from P to AP requires a bigger current than going from AP to P). Moreover, when using STT, a larger access transistor size is required, thus limiting the application density. Yet another challenge with scaling down using STT is that the thermal stability factor scales down linearly with the area and the increase in retention failures due to thermal instability results in unreliable operations [17]. STT-based applications also face problems when high write speed is required because the switching current of STT is inversely proportional to the write pulse width [17].

The MTJ behavior model is given by [18,19]:

(1) $$I_{co}=\alpha \frac{\gamma e}{\mu_{B}g}\left(\mu_{0}M_{s}\right)H_{k}V$$

(2) $$E=\frac{\mu_{0}M_{s}H_{k}V}{2},$$

where α is the magnetic damping constant, γ is the gyromagnetic ratio, *e* is the elementary charge, $\mu_{B}$ is the Bohr magneton, *g* is the spin polarization efficiency factor, $\mu_{0}$ is the permeability of free space, $M_{s}$ is the saturation magnetization, $H_{k}$ is the effective anisotropy field and *V* is the volume of the free layer. Equation (3) gives the average MTJ state switching delay time (τ) [18,19].

(3) $$\tau =\tau_{0}exp\left(\frac{E}{k_{B}T}\left(1-\frac{I}{I_{c0}}\right)\right),\;\;\mathrm{when}\;\;I<I_{c0}$$

(4) $$\frac{1}{\tau }=\left[\frac{2}{C+ln(\frac{\pi^{2}\epsilon }{4})}\right]\frac{\mu_{B}P_{ref}}{em_{m}\left(1+P_{ref}P_{free}\right)}\left(I-I_{c0}\right),\;\;\mathrm{when}\;\;I>I_{co},$$

where $\tau_{0}$ is the attempt period, $k_{B}$ is the Boltzmann constant, *T* is the temperature, *C* is Euler’s constant, ϵ is the thermal stability factor, $m_{m}$ is the magnetization moment, $P_{ref}$ and $P_{free}$ are the tunneling spin polarizations.

### 2.2. Voltage-Controlled Magnetic Anisotropy (VCMA)

Magnetoelectric effects have been studied with the aim at efficiently switching the MTJ state consuming less energy [20]. Efficient energy consumption and reduced area can be achieved if a voltage-controlled MTJ with an electric field (or a voltage) is used [21,22]. With the VCMA effect, an electric field is used in order to switch the MTJ state. It occurs by an accumulation of electron charges induced by the electric field changing the occupation of atomic orbitals at the interface. This and the spin-orbit interaction lead to a change of magnetic anisotropy [20,23,24].

**Figure 2:**
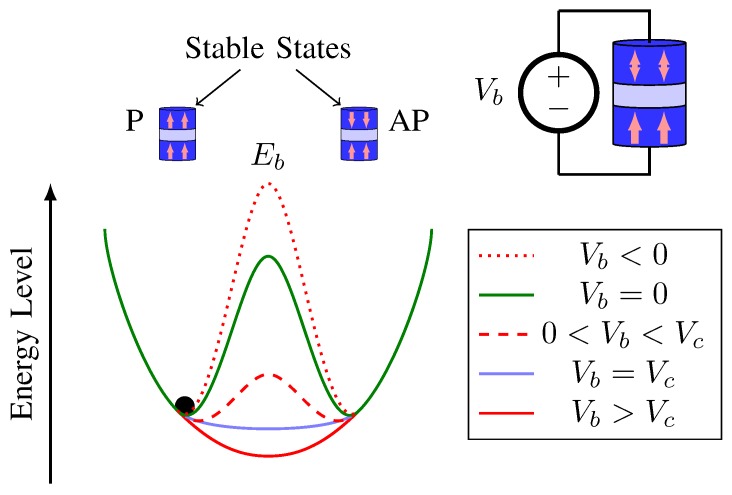
Structure and stable states of the voltage-controlled magnetic anisotropy (VCMA)-MTJ device.

Figure 2 outlines the VCMA-MTJ operational characterization. As the switching is performed through voltage, the increasing of the barrier thickness can decrease the parasitic conductance and so the effect of current-induced torques [17]. The energy barrier between the P and AP states can be reduced with the use of VCMA. Therefore, a voltage applied across the MTJ terminals facilitates the switch of its states. When the MTJ critical voltage $V_{c}$ is smaller than the switching voltage $V_{b}$, the energy barrier $E_{b}$ between two stable magnetization states can be eliminated. Equation (5) gives the minimum $V_{c}$ for successful VCMA-MTJ switching [25]:

(5) $$V_{c}=\Delta \left(0\right)k_{B}Tt_{ox}/\xi A,$$

where ξ is the VCMA coefficient to weigh the perpendicular magnetic anisotropy (PMA) change under $V_{b}$, Δ(0) is the thermal stability under zero voltage, *A* is the sectional area of the MTJ, *T* is the temperature, $t_{ox}$ is the MTJ oxide layer thickness and $k_{B}$ is the Boltzmann constant.

The VCMA-MTJ dynamics changes continuously through its unstable states, once $V_{c}$ is achieved [26]. The $E_{b}$ of the intermediate states comes back to a greater value than the stable states leading to stabilize the MTJ in its P or AP state when the excitation of its terminals has finished. Compared to STT, VCMA does not require large currents facilitating the scalability of its applications and resulting in a lower power consumption. However, practical VCMA devices face reliability issues which have to be better understood [27].

### 2.3. Spin-Orbit Torque (SOT)

With the SOT technique, three terminals are used to separate the write and read paths allowing for a symmetrical switching current between the MTJ states. With that, the possibility of a bit flip during the read operation is reduced, therefore increasing the read stability [17]. When a current crosses the non-magnetic layer, spins are accumulated and a torque switching is generated over the magnetization of the ferromagnetic layer. A faster switching can occur using SOT with the elimination of the time-demanding precessional motion [28]. However, one disadvantage of SOT is that it requires bigger cell size than STT-based applications due to its three terminal structure, so it can be not compatible with high-density applications.

## 3. Digital Applications

This sections addresses some MTJ-based digital applications.

### 3.1. Memory

One of the most known MTJ applications is the MTJ-based memory. Magnetic Random-Access Memory (MRAM) aims at combining the best characteristics of dynamic random-access memory (DRAM), static random-access memory (SRAM) and flash memory in order to become the “universal memory” [29,30]. Using the intrinsic spin of electrons as a storage unit and the difference of the MTJ resistance in its parallel and antiparallel states to represent the “0” and “1”in the binary system, MTJs are applied as the basic elements in information storage.

**Figure 3:**
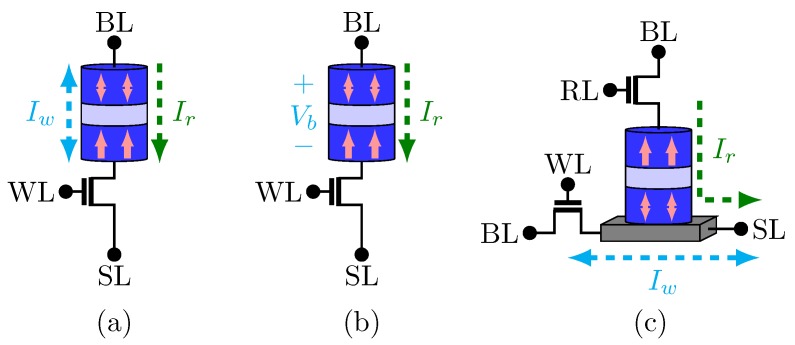
Memories devices: (**a**) STT-MRAM (**b**) VCMA-MeRAM (**c**) SOT-MRAM.

The structures of MTJ-based MRAM differ in the write operation approach and the number of MOS transistors [31,32,33,34] used to build a unit cell of memory. Figure 3a illustrates the spin transfer torque MTJ-based MRAM (STT-MRAM) bit-cell structure. Each bit-cell has only one transistor with the STT-MTJ allowing higher density memories. As it is shown in Figure 3a, while the bi-directional current $I_{w}$ is responsible for switching the MTJ state during the write operation, the MTJ state is defined comparing the read current $I_{r}$ with a reference current [15]. It is worth mentioning that STT-MRAM is characterized by an asymmetric write operation. This occurs because the current required to switch from the AP to P state is smaller than that of switching from the P to AP state [33]. Therefore, in order to achieve the requirement of worse case of the write operations, the access transistor has to be large [32,33,35].

Furthermore, STT-MRAM has intrinsic problems in terms of long latency and high write power when compared with other mechanisms [26]. On the other hand, MTJ with VCMA provides magnetization flipping upon a voltage pulse [21,36]. Using voltage for writing data into an MTJ instead of a charge current can result in a lower energy dissipation [26]. Moreover, the required driving current decreases for the write operation allowing a reduction of the access transistor size [26]. VCMA-MTJ-based memory performs better than STT-MRAM with respect to switching energy and density [20,26,37,38].

The VCMA-MeRAM structure is illustrated in Figure 3b. Just like STT-MRAM, it has 1 MTJ and 1 access transistor in series. Its write operation consists of either maintaining the MTJ state or switching it. An extra circuit is responsible for checking the MTJ state and deciding to switch or maintain the MTJ state.

Another structure is the SOT-MRAM. Its bit-cell design is represented in Figure 3c. As can be seen, its integration density capacity is reduced due to the two access transistors in each bit-cell [32,34]. In this MTJ-based MRAM, the write path and the read path are different. The write current $I_{w}$ is generated by the voltage applied between the source line (SL) and the bit line (BL). It is polarized and it switches the magnetization direction of the MTJ free layer. On the other hand, during the read operation, the MTJ state is read according to the magnitude of $I_{r}$ [39].

Some works suggest that SOT-MRAM requires a lower write time and a lower write energy than STT-MRAM [40,41,42,43]. On the other hand, the results presented in Reference [26] show that VCMA-MeRAMs outperform STT-MRAM in terms of area, speed and energy consumption. Table 1 reproduces some comparisons presented in Reference [44]. However, it should be highlighted that they are developing technologies. Even if products based in STT MTJ is already in commercialization, intensive research and development are being done in this field especially regarding VCMA and SOT.

**Figure 4:**
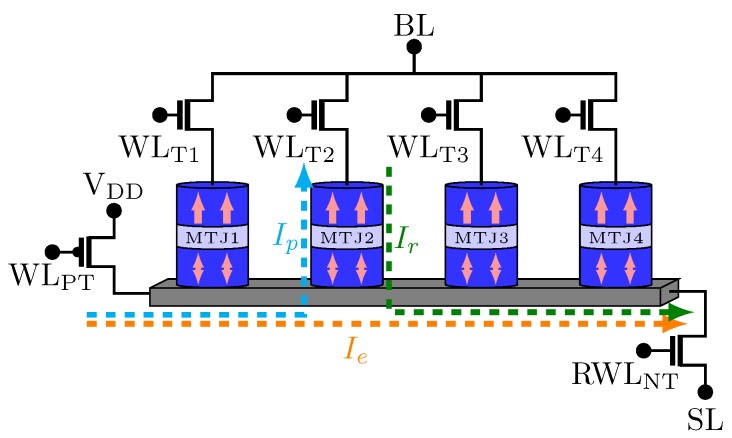
4-bit NAND-SPIN structure.

Some other memories using multiples mechanisms to write and read MTJ device have also been proposed. NAND-SPIN is an example of MTJ-based memory [32] which uses more than one of the cited switching mechanisms (see Figure 4). The idea is to take advantages of both STT and SOT mechanisms, while aiming at better performance. Compared to SOT-MRAM, NAND-SPIN memory has a better integration density. It occurs because the transistors are shared by several MTJs. On the other hand, compared to STT-MRAM, NAND-SPIN leads to better energy performance [32].

**Figure 5:**
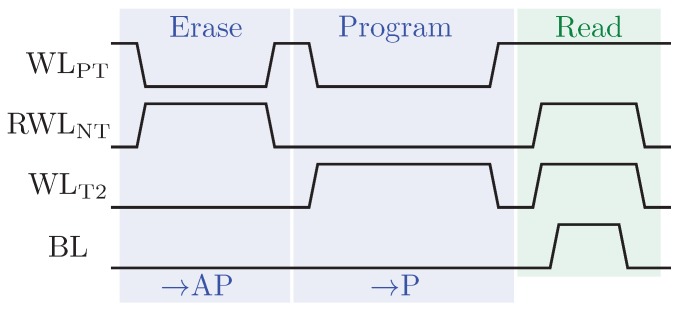
Timing diagram of write and read operations for the NAND-SPIN over MTJ2.

Figure 5 illustrates the timing diagram for the write and read operations over MTJ2. As can be noticed, the write operation of the NAND-SPIN has two phases:

- Erase: this phase initializes the MTJs at their AP states. Moreover, the transistors NT and PT are on, while the access transistors are off. Then, a write current $I_{e}$ goes through the shared metal strip.
- Program: in this phase, the transistor PT and the corresponding access transistor are on (for the case represented in Figure 5, the corresponding access transistor is T2). Therefore, a current $I_{p}$ flows through the MTJ from the free layer to the pinned layer switching the MTJ state to P by the STT mechanism.

During the read operation, the access transistor corresponding to the MTJ which will be read and the transistor NT are on. The reading of the MTJ state is made by comparing a reference current with $I_{r}$.

Even if MTJ has high tolerance to radiation [45], the MOS access transistors in MTJ-based memory structures may be impacted by radiation. Among other factors, tolerance to radiation is one important and studied field [46,47,48,49] when it is necessary to take into account performance and risks of MRAM in the integration process of MOS technology.

### 3.2. Logic Gates

Non-volatile logic gates are other MTJ applications which allow for the reduction in area and power consumption.

**Figure 6:**
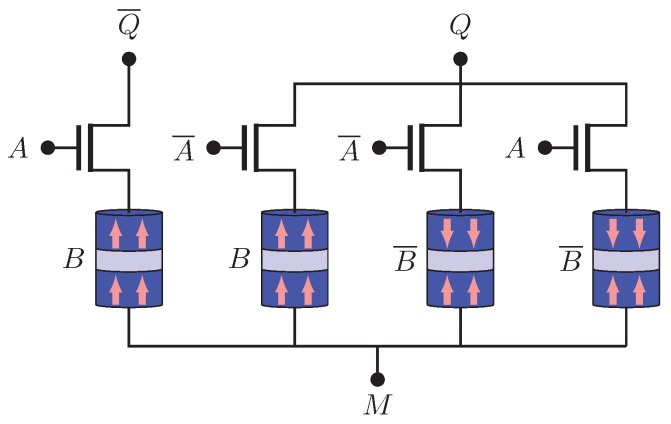
NV-AND/NV-NAND structure.

Figure 6 illustrates the NV-AND / NV-NAND structure. *Q* and $\bar{Q}$ represent AND and NAND operations, respectively. The truth table is given by Table 2 and the logic functions are illustrated by (6) and (7). This structure gives correct functions for any resistive level of MTJ.

(6) Q=AB

(7) $$\bar{Q}=\bar{AB}=\bar{A}+\bar{B}=\bar{A}B+\bar{AB}+A\bar{B}$$

**Figure 7:**
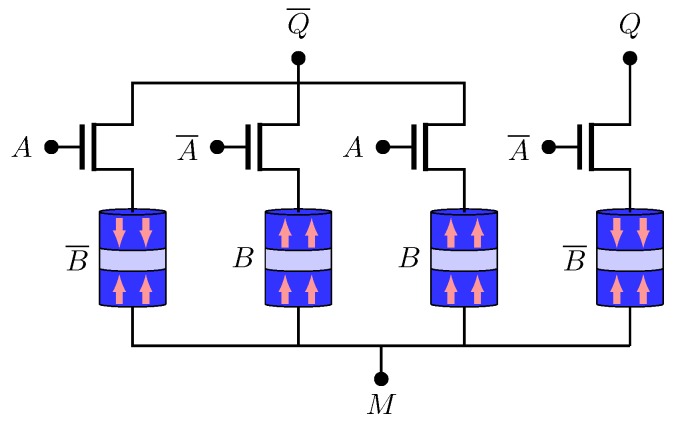
NV-OR/NV-NOR structure.

The NV-OR/NV-NOR structure is shown in Figure 7. *Q* and $\bar{Q}$ represent OR and NOR operations, respectively. The truth table is given by Table 3 and the logic functions are illustrate by (8) and (9).

(8) $$Q=\bar{A}B+A\bar{B}+AB$$

(9) $$\bar{Q}=\bar{A}.\bar{B}.$$

**Figure 8:**
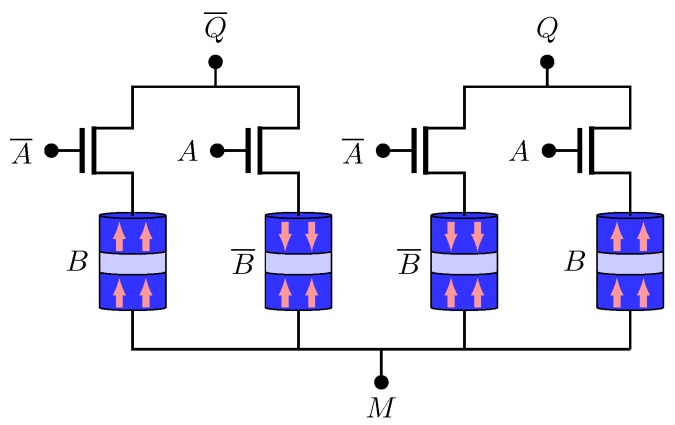
NV-XOR/NV-NXOR structure.

Figure 8 presents the NV-XOR/NV-NXOR structure. *Q* and $\bar{Q}$ represent XOR and NXOR operations, respectively. The truth table is given by Table 4 and the logic functions are illustrate by (10) and (11).

(10) $$Q=\bar{A}B+A\bar{B}$$

(11) $$\bar{Q}=\bar{A}.\bar{B}+AB$$

In Reference [50], the author proposes some logic gate structures that reduce the number of NMOS transistors and the MTJ when compared with the logic gate structures presented. However, for their proper operation, some NMOS and MTJ settings must be followed such as their resistance configurations.

### 3.3. Look-up Table (LUT)

One of the main components of FPGAs are Look-Up Tables (LUTs) which are usually composed by SRAM cells [51]. Nevertheless, SRAM-based LUTs have limitations such as low logic density, volatility and high static power [52]. On the other hand, in MTJ-based LUT only the data processing portion is active, whereas other parts are powered off reducing the power consumption and mutual disturbance.

Table 5 reproduces the comparison of some MTJ-based LUTs presented in Reference [53]. While the spin-based LUTs presented in References [53,54,55,56,57] require a clock, in Reference [52], a 6-input fracturable non-volatile Clockless LUT (C-LUT) using a spin Hall effect (SHE)-based MTJ is proposed for combinational logic operations without needing a clock. This C-LUT eliminates the sense amplifier generally employed and decreases the area compared to the STT-MTJ-based C-LUT.

### 3.4. Flip-Flop (FF)

**Figure 9:**
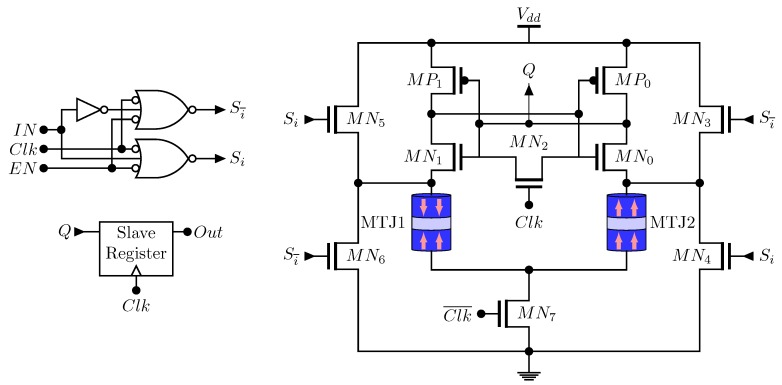
Spin-MTJ based Non-Volatile Flip-Flop proposed in Reference [59].

The loss of the data due to power failures and system crashes can be avoided using flip-flop based on non-volatile memory. In Reference [59], one of the first non-volatile flip-flop based on MTJ for FPGA and System On Chip (SoC) circuits is proposed. All the data processed is permanently stored in the Spin-MTJ memory cells making these circuits fully non-volatile. Figure 9 illustrates its full schematic, that is, the sense amplifier with the bidirectional current source.

The NOR gates control the activation of the transistors MN3, MN4, MN5, MN6. Each time, two of them are active. The signal EN enables the current source thus reducing the power dissipation as the circuit is in static mode. The signal IN writes the pair of MTJs and gives the current direction. MN7 switches between the writing and reading mode. When Clk=1, the slave register keeps the previous data and the input data is stored. On the other hand, when Clk=0, the data stored is read by the sense amplifier and the slave register updates with *Q*. Other non-volatile flip-flop implementations can be found in References [19,60,61,62].

### 3.5. Full Adder (FA)

**Figure 10:**
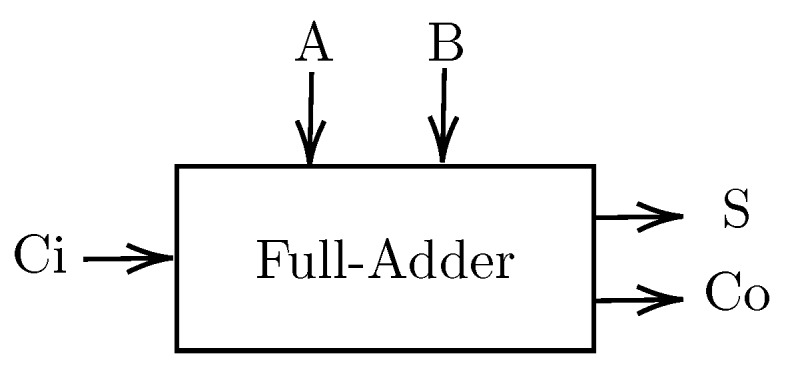
Single-bit full adder (FA) schematic.

Figure 10 illustrates a single-bit full adder structure. It consists of three inputs (*A*, *B* and $C_{i}$) and two outputs (*S* and $C_{o}$) given by (12) and (13). FA is a basic unit to an arithmetic operation in a CPU. Low-power and high-density FA are desirable.

(12) $$S=A⊕B⊕C_{i}=ABC_{i}+A.\bar{B}.\bar{C_{i}}+\bar{A}.B.\bar{C_{i}}+\bar{A}.\bar{B}.C_{i}$$

(13) $$C_{o}=AB+AC_{i}+BC_{i}$$

**Figure 11:**
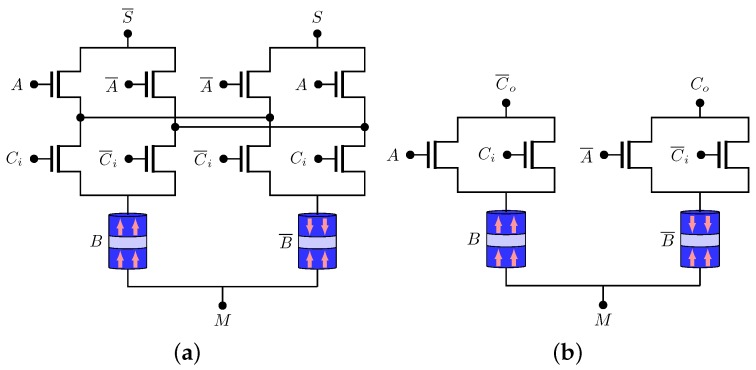
Full adder structure: (**a**) SUM sub-circuit (**b**) CARRY sub-circuit.

Figure 11 presents the SUM and the CARRY sub-circuits proposed with MTJ elements [50]. Several other non-volatile full adders were proposed in References [8,63,64,65].

## 4. Mixed and Analog Applications

This sections addresses MTJ-based mixed and analog applications.

### 4.1. Comparator

One of most important components of an ADC is the comparator. A low-power and high-speed comparator is essential to build a high-speed ADC. Traditional comparators have several stages of latches and amplifiers [66].

**Figure 12:**
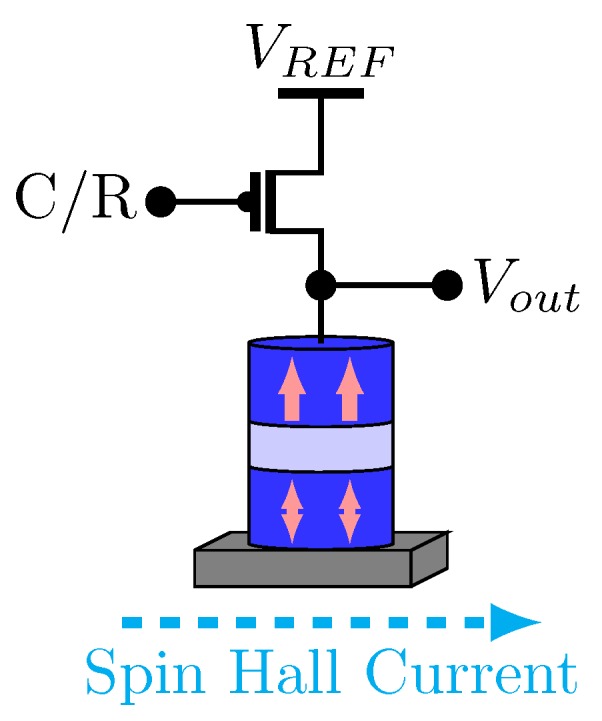
Comparator proposed in Reference [67].

In Reference [67], an MTJ comparator is proposed using one transistor and one spin Hall driven MTJ (see Figure 12). The spin Hall metal (SHM) is in contact with the free layer of the MTJ. Consequently, a spin current transversely can be produced by a charge current flowing in the SHM, hence applying spin-transfer torque on the free layer for switching.

### 4.2. Analog to Digital Converter (ADC)

The ADC poses many challenges depending on its application. For example, compact ADCs could be important in parallel data conversion for image processing [68]. Furthermore, with the downscaling of CMOS, the increased static energy consumption has to be avoided. The traditional architecture and operation mode of the ADCs are not suitable for improvement on resolution and power consumption [66,69]. New technologies and new device design are required to meet the demands of IoT devices, cognitive radios and other applications in terms of ADC constraints related to sampling rate, area, power consumption and bandwith [67]. Even if a lot of data converters are based on CMOS [70], in recent years, spintronic devices have been explored in order to save area and reduce the power consumption of the ADCs [10,67,68].

**Figure 13:**
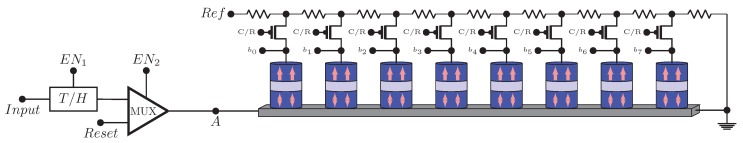
ADC proposed in Reference [67].

**Figure 14:**
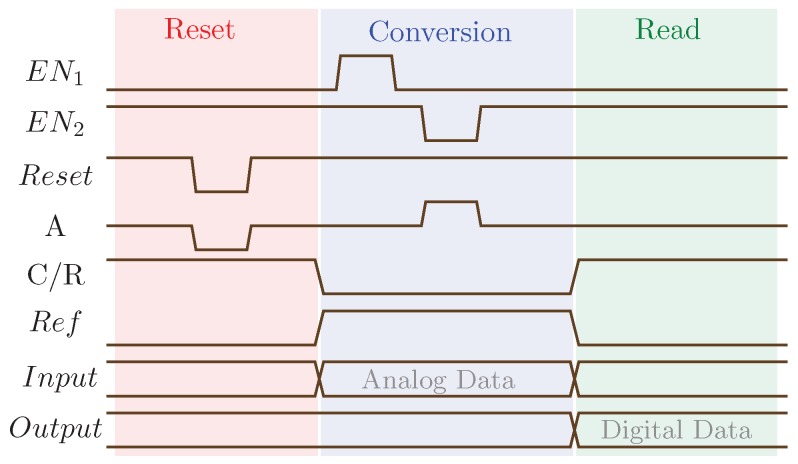
Timing diagram of the ADC operations [67].

In Reference [67], the comparator illustrated in Figure 12 is used to design a 3-bit spin-based ADC (see Figure 13). It can be noticed that 8 comparators are used to provide a 3-bit resolution. The conversion is composed of three phases: reset, conversion and read. Figure 14 shows the timing diagram of ADC operations. During the reset phase, all MTJs go to their AP state. In the conversion phase, the transistors are on. The voltages on MTJs are different due to the resistors. The VCMA effect in MTJ results in the increase of switching currents from left to right on the comparator, leading to bit levels. A pulse on EN1 samples the input signal and transmits it through the SHM, thus switching some of the MTJs. Finally, in the read phase, the transistors are off and the results are read. This ADC presents improvements in terms of power consumption compared to Flash ADC presented in the literature [67].

**Figure 15:**
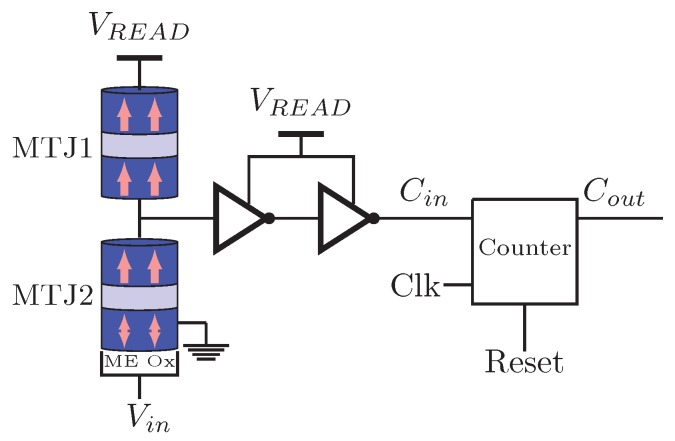
ADC proposed in Reference [10].

In order to do approximate analog to digital conversion at low voltages, a voltage-controlled stochastic switching device based on a superparamagnetic nanomagnet is proposed in Reference [10]. Figure 15 illustrates the schematic of the ADC. It is composed by a counter, two MTJs and two back-to-back inverters. As shown, the analog input enters in the ME oxide terminal of the MTJ and the counter output gives the digital output. When the input voltage increases, the probability of the MTJ2 in the AP state increases too. When the MTJ2 is in its AP state $C_{in}=1$, otherwise $C_{in}=0$. At the positive edge of the clock, the counter counts up if $C_{in}=1$. Finally, the digital count is translated to a binary code through look-up-tables. The results of Reference [10] show that the proposed ADC is suitable for sensors which require low-voltage conversions. Moreover, compact and lower power can be achieved using this proposition.

### 4.3. Non-Uniform Clock Generator

Non-uniform sampling (NUS) analog-to-information converters (AIC) generally have a non-uniform clock generator. Normally, a pseudo-random generator Linear Feedback Shift Register (LFSR) integrates an asynchronous clock generator used in non-uniform sampling techniques to randomly select a clock signal. However, this circuit can require a large number of CMOS transistors leading to significant power dissipation and area consumption.

**Figure 16:**
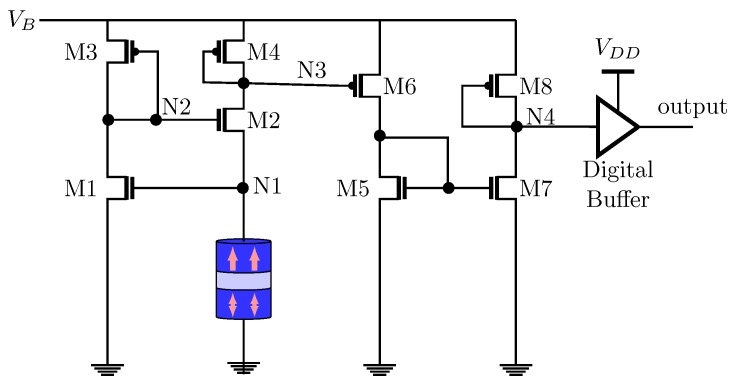
The MTJ-based voltage-controlled stochastic oscillator (VCSO) proposed in Reference [71].

In Reference [71], the authors propose a non-uniform clock using VCMA-MTJs which outperforms the CMOS-based ones in terms of area and power. Figure 16 illustrates the MTJ-based voltage-controlled stochastic oscillator (VCSO) proposed in Reference [71]. The voltage $V_{B}$ is related to the maximum frequency of an analog input signal. M1, M2 and M3 are responsible for maintaining the voltage on the N1 node independent of the variations of the MTJ resistance. With that, $V_{B}$ direct defines the voltage across the MTJ controlling its switching rate. In other words, $V_{B}$ increases if the input signal frequency is high. This will reduce the MTJ energy barrier, resulting in a higher rate of state switching. On the other hand, when the input signal frequency is low, $V_{B}$ is also low and the MTJ energy barrier continues to be high, reducing the state switching rate. The MTJ’s resistance is sensed by the amplifier. After that, a voltage variation on the N3 node related to the MTJ’s resistance fluctuation is amplified. Finally, the N4 node is sent to a buffer in which an asynchronous ADC is connected to.

It can be noticed that the amount of transistors is drastically reduced using the VCMA-MTJ instead of the traditional CMOS-based non-uniform clock generators. However, this generator considers the signal’s frequency to generate the sampling clock. Therefore, it is not suitable for signals where their frequency is unknown or those with a large bandwidth.

On the other hand, the non-uniform clock generator using MRAM-based stochastic oscillator devices called Adaptive Quantization Rate (AQR) generator proposed in Reference [72] takes into account the signal’s sparsity to generate the sampling clock. Thus, the number of samples is reduced leading to more energy savings.

**Figure 17:**
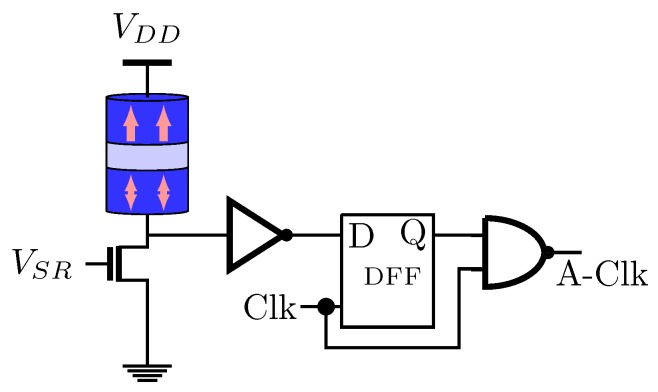
Adaptive Quantization Rate (AQR) generator proposed in Reference [72].

Figure 17 illustrates the AQR. The stochastic behavior of the VCMA-MTJ provides the non-uniform clock generation capability. The voltage $V_{SR}$ is related to the signal’s sparsity that can be known or can be estimated before. Therefore, the $V_{SR}$ applied to the NMOS of the AQR will generate a stochastic bit-stream by the MRAM-based stochastic oscillator device [72]. The Asynchronous Clock (A-Clk) is given by the result of the NAND gate between the actual clock and the output of the D-Flip-Flop (D-FF). It can be noticed that using AQR, large LFSR circuits which has many multiplexers, logic gates and D-FFs are avoided.

### 4.4. Adaptive Intermittent Quantizer (AIQ)

In Reference [9], the authors propose a Spin-based Adaptive Intermittent Quantizer (AIQ) in order to have an adaptive signal sample and quantization. Compressive sensing (CS) theory and spin-based devices are applied for energy-aware acquisition of spectrally sparse signals. Compared to conventional CMOS designs, the use of VCMA-MTJ allows reducing the energy consumption via an instant on/off operation. It does not require the use of a backing store and it leads to a fast sampling rate (SR), an adaptive quantization resolution (QR) and area reduction.

**Figure 18:**
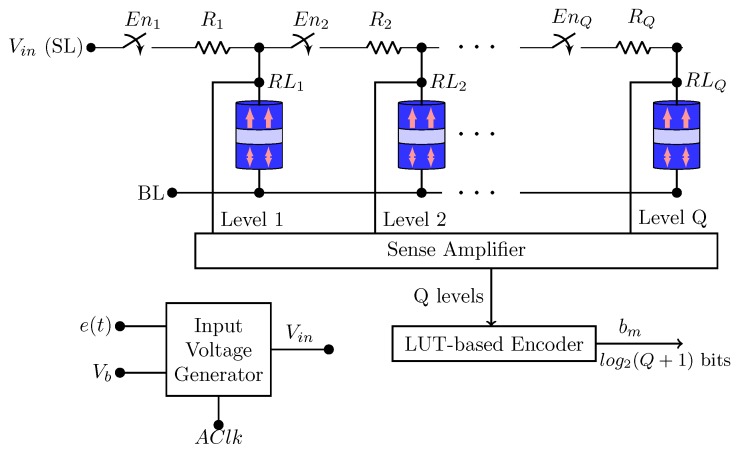
Q-level adaptive intermittent quantizer (AIQ) architecture proposed in Reference [9].

Figure 18 shows the Q-level AIQ architecture proposed in Reference [9], where Q is the number of QR levels. The quantization levels of the AIQ are given by changes in the energy barrier of the VCMA-MTJs.

There are three main steps during the AIQ operation [9]:

- Reset: all active VCMA-MTJs go to their parallel state, that is, they are reset to zero. In order to do this, the source line (SL) is set to “0”, bit line (BL) is set to “1” and read lines (RLs) are in high impedance.
- Sampling: the active VCMA-MTJs are written. In other words, the energy barrier of the active VCMA-MTJs are modified and set by the bias voltage $V_{b}$ applied across the active VCMA-MTJs followed by the analog input e(t). The sampling rate of e(t) is controlled by the Adaptive Clock (AClk). In addition, SL is set to $V_{in}$, BL is set to “0” and RLs are in high impedance.
- Read or Sensing: in this step, the sense amplifier reads the data stored in each VCMA-MTJ. SL is in high impedance and BL is set to “0”.

The switches and resistors presented in Figure 18 are responsible for the adaptive quantization resolution levels. With the resistors, different MTJs have different $V_{b}$. Thus, while some MTJs require lower input voltages to turn on; others switch their state only with higher input voltages. The switches allow for the optimization of the QR by turning off the MTJs which are not used.

In Reference [9], the authors used 255 VCMA-MTJs to realize a range of quantization resolutions from 1-bit to 8-bit ADC operations. Their obtained results show that this AIQ leads to better power consumption compared to other CS ADC designs.

### 4.5. Sensors

**Figure 19:**
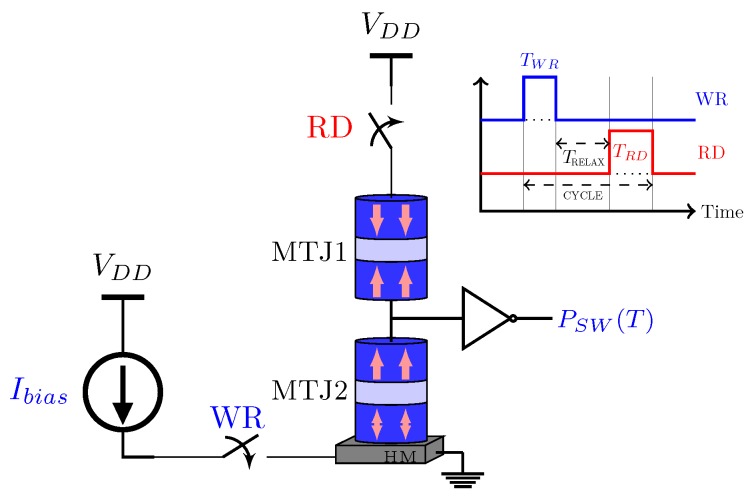
The MTJ-based temperature sensor proposed in Reference [73].

With the increase of transistor scaling and power density, the temperature monitoring and the analysis of heating effects are very important, leading temperature sensors to become a relevant component in SoC. MTJ can also be used as a sensor resulting in high conversion, compact and low energy consumption devices. In Reference [73] an MTJ-based temperature sensor is proposed (see Figure 19). The SOT switching mechanisms are used to stochastically switch the MTJ state according to the operating temperature in the presence of thermal noise. The MTJ probabilistic switching characteristics of the MTJ can be given by the Landau-Lifshitz-Gilbert (LLG) equation [73]:

(14) $$\frac{d\hat{m}}{dt}=-\gamma \left(\hat{m}\times H_{eff}\right)+\alpha \left(\hat{m}\times \frac{d\hat{m}}{dt}\right)+\frac{1}{qN_{s}}\left(\hat{m}\times I_{s}\times \hat{m}\right),$$

where $\hat{m}$ is the vector of free layer magnetization, $\gamma =\frac{2\mu_{B}\mu_{0}}{h}$ is the gyromagnetic ratio for electron, $\mu_{B}$ is Bohr magneton, $H_{eff}$ is the effective magnetic field including the shape anisotropy field for elliptic disks, α is Gilbert’s damping ratio, $N_{s}=\frac{M_{s}V}{\mu_{B}}$ is the number of spins in a free layer of volume *V*, $M_{s}$ is saturation magnetization and $I_{s}$ is the spin current generated by the heavy metal layer.

As can be seen in Figure 19, the sensor MTJ (MTJ2) and the reference MTJ (MTJ1—fixed at its AP state) form a voltage divider circuit. The switching probability ($P_{SW}\left(T\right)$) is given by the inverter output. The control signals RD and WR active the read and write current paths respectively. In the write operation, WR is on and then the $I_{bias}$ current probabilistically switches the magnet depending on the temperature. On the other hand, during the read operation, RD is on and the sensor MTJ final state is determined. Between the write and the read operations, there is a “relaxation” period ($T_{RELAX}$) in order to stabilize the magnetization directions after the write operation. This MTJ-based temperature sensor achieves better results than state-of-the-art CMOS temperature sensors in terms of throughput and energy consumption [73].

Regarding a frequency sensor, Reference [74] proposes an MTJ-based microwave detector that can be used, for example, as a spectrum analyzer. The ferromagnetic resonance in MTJs can determine the microwave frequency. With this, the mixer circuit is not required in conventional RF diode detection.

**Figure 20:**
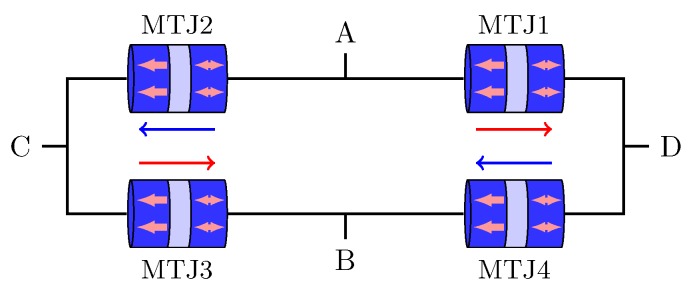
Full bridge configuration.

In order to monitor at the IC level, MTJ full Wheatstone bridges are proposed in Reference [75]. As can be seen in Figure 20, the currents in MTJ1 and MTJ3 flows in the opposite direction than it does in MTJ2 and MTJ4. Therefore, depending on the sign of the current through terminals A and B, the resistance of MTJ1 and MTJ3 increases/decreases, while MTJ2 and MTJ4 change in the opposite way, leading to the Wheatstone bridge operation.

As we have shown, several papers have addressed MTJ applications. However, there are still many other domains where MTJ can be applied. Using, for example, its stochastic behavior, MTJ can also be used as a memristive probabilistic device and to implement the activation function [76] for Spiking Neural Networks (SNNs). As SNN intends to mimic the computational efficiency of the human brain, non-volatile resistive memories can be found in the literature to mimic a stochastic one-bit synapse [76,77]. The MTJ conductance is used to modulate the voltage spike produced by a pre-neuron resulting in a post-synaptic current.

## 5. Conclusions

The characteristics of MTJs make this type of device suitable in various areas. This paper provided a review of several works where MTJs were used to improve circuit consumption, area and efficiency. The text showed, in particular, that MTJ has been considered a promising alternative for the development of universal memory. Furthermore, the reported works show that MTJ can also play an important role in several other fundamental blocks such as comparators, ADC and sensors.

The use of MTJ is quite interesting in many applications where it can replace multiple transistors. These transistors, at nanometers scale, face problems of increasing static power consumption. Unfortunately, MTJ is a technology that still requires a great deal of development and only a few companies produce it. The simultaneous control of several MTJ parameters is one of the challenges of large-scale use of MTJ-based devices, for example, maintaining simultaneously high thermal stability and low switching current. Moreover, there are reliability challenges involving write and read failures. Indeed, process variations can significantly influence bit errors. On the other hand, scaling to smaller cell sizes is difficult, due to several factors such as the magnitude of the required switching currents. Despite these difficulties, the simulation results are promising regarding the advantages of using MTJ. In addition, new effects related to the switching of the magnetic state of MTJ devices have been studied and more optimal materials to be used in the MTJ structure have been developed.

## Figures and Tables

**Table 1:** Comparison between some MTJ-based memories.

	STT-MRAM	SOT-MRAM	VCMA-MeRAM
Read Time (ns)	1–5	1–5	1–5
Write Time (ns)	5–10	<1	<1
Cell Size (area in $F^{2}$)	40–50	50–70	20–30
Bit Density (Gb/cm^2^)	1	0.75	2
Read Energy/Bit (fJ)	10–20	10–20	1–5
Write Energy/Bit (fJ)	100–200	<10	<5

**Table 2:** Truth table of AND/NAND.

A	B	*Q* (AND)	$\bar{Q}$ (NAND)
0	0	0	1
0	1	0	1
1	0	0	1
1	1	1	0

**Table 3:** Truth table of OR/NOR.

A	B	*Q* (OR)	$\bar{Q}$ (NOR)
0	0	0	1
0	1	1	0
1	0	1	0
1	1	1	0

**Table 4:** Truth table of XOR/NXOR.

A	B	*Q* (XOR)	$\bar{Q}$ (NXOR)
0	0	0	1
0	1	1	0
1	0	1	0
1	1	0	1

**Table 5:** Characteristics of LUT designs.

Design	Write/Read Operation	Features and Challenges
FIMS-LUT	Magnetic	High Speed
[58]	Field/TMR	High Power Consumption
		High Area Overhead
TAS-LUT	Magnetic	Relatively High Speed
[54]	Field/TMR	High Power Consumption
		Medium Area Overhead
STT-LUT	STT/TMR	High Speed
[53]		Low Power Consumption
		Low Area Overhead
A-LUT	STT/TMR	High Speed
[53]		Scalable Power Consumption
		Low Area Overhead

## Abbreviations

The following abbreviations are used in this manuscript:

ADC	Analog to Digital Converter
AIC	Analog-to-Information Converter
AIQ	Adaptive Intermittent Quantizer
AP	Anti-parallel
AQR	Adaptive Quantization Rate
BL	Bit-line
CMOS	Complementary Metal Oxide Semiconductor
CPU	Central Processing Unit
CS	Compressive Sensing
DRAM	Dynamic Random-Access Memory
FA	Full Adder
FF	Flip-Flop
FL	Free Layer
FPGA	Field Programmable Gate Array
GMR	Giant Magnetoresistance
HM	Heavy Metal
IC	Integrated Circuits
IoT	Internet of Things
LFSR	Linear Feedback Shift Registe
LUT	Look-up Table
MRAM	Magnetic Random Access Memory
MTJ	Magnetic tunnel junction
NUS	Non-Uniform Sampling
NVM	Non-Volatile Memory
P	Parallel
PL	Pinned Layer
PMA	Perpendicular Magnetic Anisotropy
QR	Quantization Resolution
RL	Read Line
SET	Single-Event Transient
SHM	Spin Hall Metal
SL	Source Line
SoC	System On Chip
SR	Sampling Rate
SRAM	Static Random-Access Memory
STT	Spin Transfer Torque
SOT	Spin-Orbit Torque
TMR	Tunnel Magnetoresistance
VCMA	Voltage-Controlled Magnetic Anisotropy
VCSO	Voltage-Controlled Stochastic Oscillator
WL	Word-line
